# The correlation analysis between the Oxford classification of Chinese IgA nephropathy children and renal outcome - a retrospective cohort study

**DOI:** 10.1186/s12882-020-01913-7

**Published:** 2020-07-01

**Authors:** Heyan Wu, Zhengkun Xia, Chunlin Gao, Pei Zhang, Xiao Yang, Ren Wang, Meiqiu Wang, Yingchao Peng

**Affiliations:** 1 Department of Pediatrics, Jinling Hospital, The First School of Clinical Medicine, Southern Medical University, Nanjing, China; 2 Department of Pediatrics, Nanfang Hospital, Southern Medical University, Guangzhou, China; 3 Department of Pediatrics, Jinling Hospital, Nanjing Medical University, Nanjing, China

**Keywords:** IgA nephropathy, Oxford classification, Children, Renal outcome

## Abstract

**Background:**

The 2016 Oxford Classification’s MEST-C scoring system predicts outcomes in adults with IgA nephropathy (IgAN), but it lacks tremendous cohort validation in children with IgAN in China. We sought to verify whether the Oxford classification could be used to predict the renal outcome of children with IgAN.

**Methods:**

In this retrospective cohort study, 1243 Chinese IgAN children who underwent renal biopsy in Jinling Hospital were enregistered from 2000 to 2017. The combined endpoint was defined as either a ≥ 50% reduction in estimated glomerular filtration rate (eGFR) or end-stage renal disease (ESRD). We probed into the relevance betwixt the Oxford classification and renal prognosis.

**Results:**

There were 29% of children with mesangial proliferation(M1), 35% with endocapillary proliferation (E1), 37% with segmental sclerosis/adhesion lesion (S1), 23% with moderate tubular atrophy/interstitial fibrosis (T1 25–50% of cortical area involved), 4.3% with severe tubular atrophy/interstitial fibrosis (T2 > 50% of cortical area involved), 44% with crescent in< 25% of glomeruli(C1), and 4.6% with crescent in> 25% of glomeruli (C2). All children were followed for a medial of 7.2 (4.6–11.7) years, 171 children (14%) arrived at the combined endpoint. The multivariate COX regression model revealed that the presence of lesions S (HR2.7,95%CI 1.8 ~ 4.2, *P*<0.001) and T (HR6.6,95%CI 3.9 ~ 11.3, *P*<0.001) may be the reason for poorer prognosis in the whole cohort. In contrast, C lesion showed a significant association with the outcome only in children received no immunosuppressive treatment.

**Conclusions:**

This study revealed that S and T lesions were useful as the long-term renal prognostic factors among Chinese IgAN children.

## Background

IgA nephropathy (IgAN), being particularly frequent in Asia, is the primary reason for end-stage renal disease (ESRD) in all ages [1]. Since IgAN does not exhibit a specific serologic profile, a percutaneous kidney biopsy remains a definitive tool to establish the diagnosis of IgAN [2]. Additionally, the prognostic value of histological data has become increasingly recognized in the past decade [3]. As a new pathological classification standard to judge the renal prognosis of IgAN, the Oxford classification [4] has been put forward in recent years. The purpose of the Oxford classification was to consider the pathological features associated with clinical outcomes independently of clinical data and to improve the current ability to predict outcomes in IgAN patients. Although the classification standard has been formulated by rigorous methodology, its clinical application in children needs to be further verified. What’s more, IgAN has regional and ethnic differences, which determines that the Oxford classification needs to be refined in different populations and races. In this study, the clinical and pathological data of IgAN followed up for a long time in our department were retrospectively analyzed to assess the predictability of Oxford classification among Chinese children.

## Methods

### Inclusion criteria and clinical data set

Children with IgAN who underwent renal biopsy in Jinling Hospital were enrolled from January 1, 2000, to December 31, 2017, in this retrospective cohort study. The inclusion criteria were follow-up ≥12 months, age at the time of biopsy ≤18 years, a minimum of eight glomeruli and an initial eGFR≥15 ml/min /1.73m^2^. The exclusion criteria were secondary IgAN caused by Henoch-Schonlein purpura, liver cirrhosis, systemic lupus erythematosus, hepatitis B virus infection, tumour, ankylosing spondylitis and psoriasis. The following clinical data including age, gender, duration from onset to renal biopsy, estimate glomerular filtration rate (eGFR), mean arterial pressure (MAP), proteinuria and therapeutic regimen [Renin angiotensin aldosterone system blockade (RASB), glucocorticoid (GC) and other immunosuppressant agents] were collected during biopsy and follow-up. Immunosuppressive therapy after kidney biopsy was defined as treatment with any immunosuppressive agent, regardless of duration or dose.

### Definitions

eGFR was calculated using the Schwartz formula [5], and the CKD-EPI equation [6] was used in adolescents aged > 16 years at the time of biopsy. ESRD was defined as eGFR < 15 mL/min/1.73m^2^, initiation of dialysis or transplantation. Survival time was defined from the time of biopsy until the date of the last follow-up, the incidence of the event of interest, or March 2019 (end of the study). The combined endpoint was defined as either ≥50% reduction eGFR or ESRD or death.

### Pathology review

Renal pathology was scored according to the Oxford classification of IgAN [4], assessed by the following parameters: mesangial hypercellularity (M), scored as absent (M0) or (M1) if≥50% of glomeruli had more than three cells per mesangial area; endocapillary hypercellularity (E), scored as E0 if absent or E1 if present; segmental glomerulosclerosis or adhesion (S), scored (S0) if absent or (S1) if present; tubular atrophy/interstitial fibrosis(T), scored as T0 (0–25% of cortical area), T1 (26–50% of cortical area), or T2(>50% of cortical area); cellular/ fibrocellular crescents scored as C0 (no crescents), C1 (crescents in at least one but < 25% of glomeruli), or C2 (crescents in more than 25% of glomeruli). Immunofluorescence studies were performed (IgG, IgA, IgM, C3) and showed at least 1+ (on scale from 0 to 3+) mesangial deposition of IgA, with IgA being the dominant immunoglobulin deposited in the glomeruli. The intensity of deposits as determined via immunofluorescence microscopy was graded semi-quantitatively on a scale from 0 to 3+, where 0 = no, 1+ = slight, 2+ = moderate, and 3+ = intense. At least two pathologists blinded to patient outcomes at the time of review confirmed the pathological results.

### Statistical analyses

Continuous variables were presented as mean ± standard deviation (normally distributed variables) or median with interquartile range (IQR, non-normally distributed variables) for data distribution. Categorical variables are presented as percentages. The Kruskal-Wallis test and chi-squared test were used to compare continuous and categorical variables, respectively. The Kaplan–Meier curve was used to illustrate univariate differences between groups of pathological variables, and the log-rank test were used to test the two curves differences. The Cox proportional hazards regression model was conducted on univariable and multivariable analyses of Oxford classification in the IgAN children. Univariable Cox regression analysis was performed for each pathological variable. Multivariable Cox regression analysis (Backward: LR approach) adjusting for other clinically essential factors including initial eGFR, initial mean arterial pressure, and initial proteinuria was performed to appraise the function of MEST-C scoring system on the renal outcome. Hazard ratio (HR) with 95% confidence interval (CI) for each variable was estimated. All probabilities were two-tailed, and *P*-value< 0.05 was considered statistically significant.

Data analysis was executed using SPSS for windows version 26 (IBM Corporation, Armonk, NY).

## Results

### Clinical features

We enrolled 1243 Chinese children diagnosed with primary IgAN from 2000 to 2017 in Jinling Hospital. Table 1 outlines the clinical, treatment and follow-up characteristics of the study children. The average age of children at diagnosis was 14 ± 4 years, with male-dominated (68%). The average value of MBP was 89 ± 16 mmHg, initial proteinuria was 0.6 (interquartile ranges, 0.3–1.4) g/day per 1.73m^2^, and the initial eGFR was 102 ± 20 ml/min per 1.73m^2^. All children were followed for a medial of 7.2 (4.6–11.7) years, 171 children (14%) arrived at combined endpoint (ESRD, *n* = 82;≥50% eGFR decline, *n* = 89). During the follow-up period, 70% of children were treated with RASB, 45% were treated with GC, and 19% received GC combined other immunosuppressive drugs.

**Table 1:** Baseline and follow-up characteristics (*n* = 1243)

At renal biopsy	Values at renal biopsy
Male, %	68
Age, years	14 ± 4
Duration from onset to renal biopsy (months)	12.0 (0.8,96.5)
eGFR, ml/min per 1.73 m^2^	102 ± 20
MAP, mmHg	89 ± 16
Proteinuria, g/day per 1.73m^2^	0.6 (0.3–1.4)
Follow-up parameters	Values during follow-up
Length of follow-up, years	7.2 (4.6–11.7)
RASB, %	70
Any immunosuppression, %	64
GC, %	45
GC + IS, %	19
Combined event, %	14
ESRD, %	6.6
50% reduction in initial eGFR,%	7.2

Values are expressed as mean ± SD; medians (interquartile ranges), or percentages

*Abbreviations*: *eGFR* Estimate glomerular filtration rate, *MAP* Mean arterial pressure, *RASB* Renin angiotensin aldosterone system blockade, *GC* Glucocorticoid, *IS* Other immunosuppressant, *ESRD* End-stage of renal disease

### Pathological findings

**Fig. 1:**
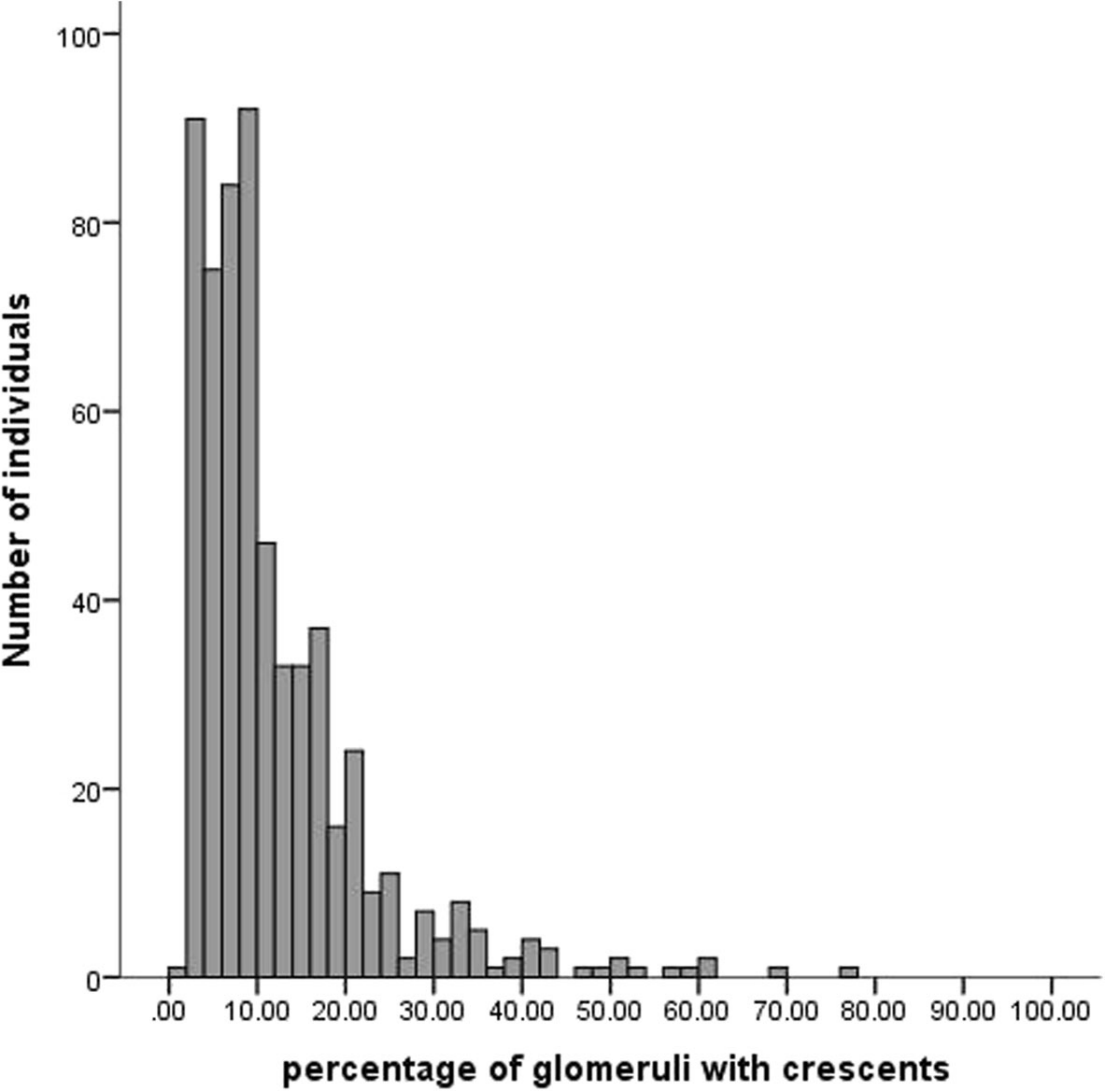
Distribution of the percentage of glomeruli with crescents in biopsies with any crescents. Crescents were present in 599(48%) of 1243 total biopsies

The average of glomeruli was 20.4 ± 4.7 per biopsy. Based on Oxford classification,29% of the children showed M1, 35% showed E1, 37% showed S1, 23% showed T1, 4.3% showed T2, 44% showed C1 and 4.6% showed C2 (Table 2). The distribution of the percentage of crescents observed in every child was shown in Fig. 1. Of 48.6% children with any cellular/ fibrocellular crescents, 28% had crescents in<10% of glomeruli, whereas 9.4% had a fraction of glomeruli with crescents one-tenth or more, 6.6% had a fraction of glomeruli with crescents one-sixth or more, and only 4.6% had a fraction of glomeruli with crescents one-fourth or more. The percentage of immunoglobulins deposited only in the mesangial region was 68%, while 32% of immunoglobulins were deposited in both the mesangial and capillary loop regions. 25% of children showed positive glomerular staining for IgG, 44% showed positive glomerular staining for IgM, 84% showed positive glomerular staining for C3, and 1.1% showed positive glomerular staining for C4. The immunofluorescence intensity of IgA was between 1+ and 3+, including 5.6% of 1+, 13% of 2+ and 81% of 3 + .

**Table 2:** Pathological findings at the time of biopsy in children with IgA nephropathy (*n* = 1243)

Pathology findings	Values at renal biopsy
The number of glomeruli per biopsy	20.4 ± 4.7
MEST-C score	% of total biopsies
M1	29
E1	35
S1	37
T1	23
T2	4.3
C1	44
C2	4.6
Deposition site of immunoglobulins	% of total biopsies
Pure-mesangium	68
Mesangium+ capillary loop	32
Immunoglobulins deposits	% of total biopsies
Glomerular IgG deposition	25
Glomerular IgM deposition	44
Glomerular C3 deposition	84
Glomerular C4 deposition	1.1
Intensity of IgA deposits ^a^	% of total biopsies
1+	5.6
2+	13
3+	81

Values are expressed as mean ± SD or number (percentage); The intensity of IgA deposits as determined via immunofluorescence microscopy was graded semi-quantitatively on a scale from 0 to 3+, where 0 no, 1 + = slight, 2 + = moderate, and 3 + = intense

*Abbreviations*: *M1* Mesangial hypercellularity>0.5, *E1* Presence of endocapillary hypercellularity, *S1* Presence of segmental glomerulosclerosis, *T1* Tubular atrophy/ interstitial fibrosis 26–50% of cortical area, *T2* Tubular atrophy/interstitial fibrosis≥50% of cortical area, *C1* Crescents in at least one but < 25% of glomeruli, *C2* Crescents in more than 25% of glomeruli

### Effects of different kidney biopsy time on the variables in Oxford classification

The median time (12 months) of onset to renal biopsy was selected as the cut-off point to analyze the effect of biopsy time on variables in the Oxford classification From Table 3*.* It showed that when the time of onset to renal biopsy was less than 12 months, the patient’s lesions were milder, dominated by S0(χ^2^ = 354.5, *P*<0.001), T0 (χ^2^ = 323.3, *P*<0.001), and C0(χ^2^ = 437.6, *P*<0.001). On the contrary, when the time of onset to renal biopsy was longer than 12 months, the lesions were corrected mainly by S1, T1–2 and C1–2. Concerning E and M lesions, there was no significant difference in time from onset to renal biopsy within available data.

**Table 3:** Comparison of all kinds of lesions with different time of onset to renal biopsy (*n* = 1243)

Variables	Time from onset to renal biopsy		χ^2^	*P*-value
	≤ 12 months	>12 months		
M0/M1	449/172	433/189	1.1	0.296
E0/ E1	411/210	401/221	0.4	0.525
S0/ S1	554/67	235/387	354.5	<0.001
T0/ T1/ T2	595/21/5	315/259/48	323.3	<0.001
C0/ C1/ C2	506/104/11	138/438/46	437.6	<0.001

*Abbreviations*: *M0* Mesangial hypercellularity≤0.5, *M1* Mesangial hypercellularity>0.5, *E0* Absence of endocapillary hypercellularity, *E1* Presence of endocapillary hypercellularity; *S0* absence of segmental glomerulosclerosis, *S1* Presence of segmental glomerulosclerosis, *T0* Tubular atrophy/ interstitial fibrosis 0–25% of cortical area, *T1* Tubular atrophy/ interstitial fibrosis 26–50% of cortical area, *T2* Tubular atrophy/interstitial fibrosis≥50% of cortical area, *C0* Absence of crescents, *C1* Crescents in at least one but < 25% of glomeruli, *C2* Crescents in more than 25% of glomeruli

### Associations between clinical and histologic variables

Linear regression analysis of Oxford classification with the most robust indicators for estimating renal decline (eGFR, MAP and proteinuria) was performed to explore the correlation between Oxford classification and clinical signs. As shown in Table 4, Children with S1, T1–2 and C1–2 lesions were associated with a reduced initial eGFR at the time of biopsy. All histological lesions (M1, E1, S1, T1–2, and C1–2) were associated individually with higher initial proteinuria at the time of biopsy. All histological lesions were associated with more upper initial MAP at the time of biopsy.

**Table 4:** Linear Regression Analysis of Oxford Classification and Clinical Indicators at Renal Biopsy

Clinical indicators	MAP (mmHg)		eGFR (ml/min/1.73m^2^)		Proteinuria (g/day/1.73m^2^)	
	R	*P*-value	R	*P*-value	R	*P*-value
M1	0.342	<0.001	0.044	0.133	0.569	<0.001
E1	0.338	<0.001	−0.331	0.389	0.527	<0.001
S1	0.541	0.042	−0.744	0.007	0.604	<0.001
T1–2	0.532	<0.001	−0.578	<0.001	0.689	<0.001
C1–2	0.549	0.008	−0.447	<0.001	0.447	<0.001

Linear Regression results are results from separate models for each independent variable

*Abbreviations*: *MAP* Mean arterial blood pressure, *eGFR* Estimate glomerular filtration rate, *M1* Mesangial hypercellularity>0.5, *E1* Presence of endocapillary hypercellularity, *S1* Presence of segmental glomerulosclerosis, *T1* Tubular atrophy/ interstitial fibrosis 26–50% of cortical area, *T2* Tubular atrophy/interstitial fibrosis≥50% of cortical area, *C1* Crescents in at least one but < 25% of glomeruli, *C2* Crescents in more than 25% of glomeruli

### Renal survival IgAN children according to Oxford classification

**Fig. 2:**
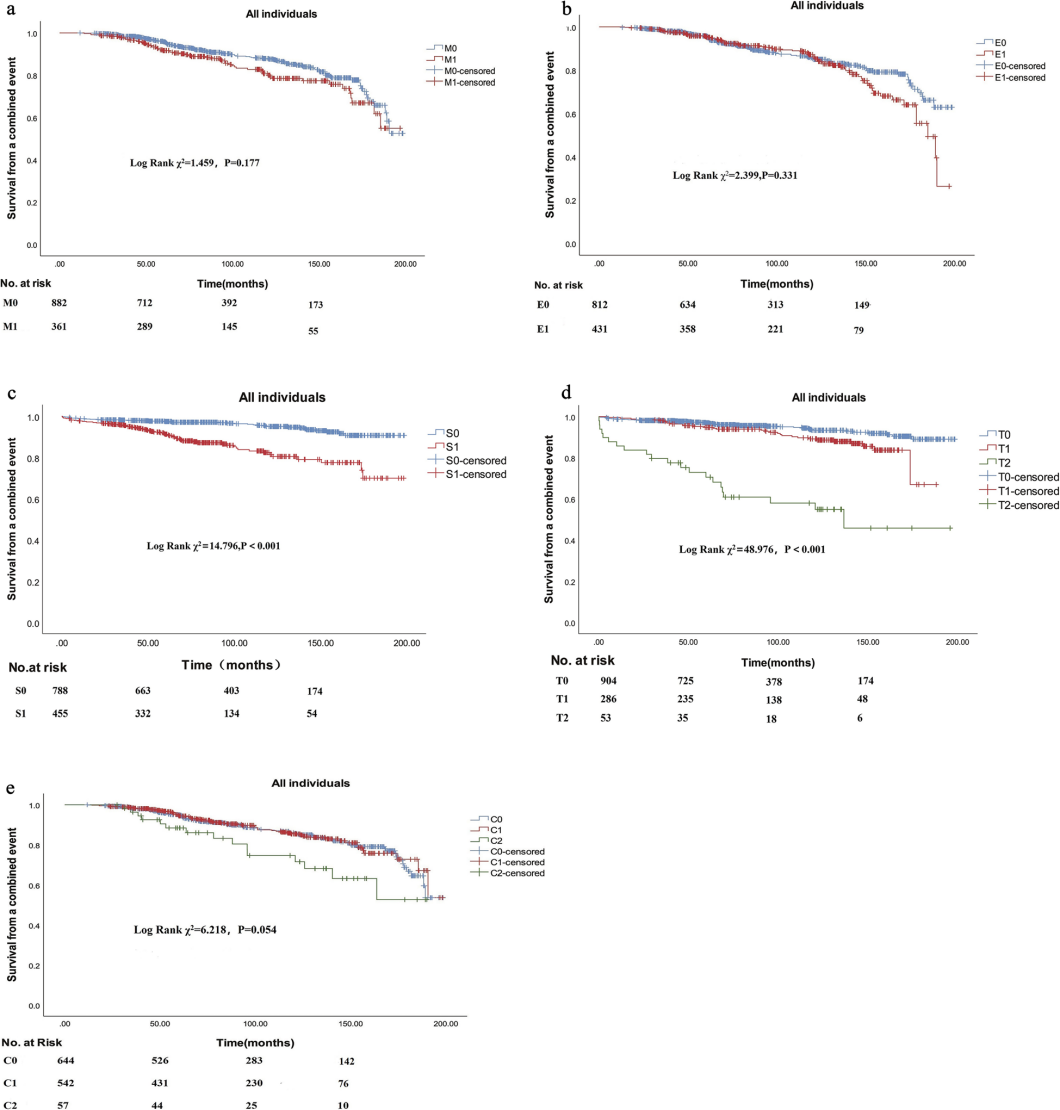
Renal survival according to pathological variables. **a** Effect of the presence of mesangial hypercellularity on survival from a combined event in all patients. **b** Effect of the presence of endocapillary hypercellularity on survival from a combined event in all patients. **c** Effect of the presence of segmental sclerosis on survival from a combined event in all patients. **d** Effect of the presence of interstitial fibrosis/tubular atrophy on survival from a combined event in all patients. **e** Effect of the presence of cellular/fibrocellular crescents on survival from a combined event in all patients

As presented in Fig. 2, the Kaplan-Meier analyses revealed that lesion S (Log-Rank, χ^2^ = 14.796, *P*<0.001; Fig. 2c) and T (χ^2^ = 48.976, *P*<0.001; Fig. 2d) were each correlation with renal survival. However, lesions M (χ^2^ = 1.459, *P* = 0.177, Fig. 2a), E (χ^2^ = 2.399, *P* = 0.331, Fig. 2b) and C (χ^2^ = 6.218, *P* = 0.054, Fig. 2e) were not associated with renal outcome.

### Associations between pathologic features and renal outcome

The associations between pathological features and renal outcomes were analyzed in a COX regression model (Table 5). With univariate COX regression model, renal outcome from a combined event were both associated significantly with lesions S (HR3.5, 95%CI 2.3 ~ 5.3, *P*<0.001), T (HR 2.6, 95%CI 2.1 ~ 3.3, *P*<0.001) and C (HR 2.1, 95%CI 1.5 ~ 2.8, *P*<0.001). However, the lesion of M (HR 1.8, 95%CI 1.3 ~ 2.3, *P* = 0.115), and E (HR 1.4, 95%CI 0.9 ~ 2.1, *P* = 0.326) were not associated with renal outcome. When adjusted for clinically essential data in the multivariate COX regression model, only S (HR2.7, 95%CI1.8 ~ 4.2, *P*<0.001) and T (HR6.6, 95%CI 3.9 ~ 11.3, *P*<0.001) lesions remained as independent predictors of renal outcome.

**Table 5:** Factors at biopsy influencing renal outcome from ESRD or 50% drop in eGFR by univariate and multivariate Cox regression

Risk factors	Univariate Cox Regression	Multivariate Cox Regression
	HR(95%CI)	HR(95%CI)
Mesangial hypercellularity		
M0	1	
M1	1.8 (1.3 ~ 2.3)	
*P*-value	0.115	
Endocapillary hypercellularity		
E0	1	
E1	1.4 (0.9 ~ 2.1)	
*P*-value	0.326	
Segmental glomerulosclerosis		
S0	1	1
S1	3.5 (2.3 ~ 5.3)	2.7 (1.8 ~ 4.2)
*P*-value	<0.001	<0.001
Tubular atrophy/interstitial fibrosis		
T0	1	1
T1 or T2	2.6 (2.1 ~ 3.3)	6.6 (3.9 ~ 11.3)
*P*-value	<0.001	<0.001
Crescent		
C0	1	1
C1 or C2	2.1 (1.5 ~ 2.8)	1.8 (1.2 ~ 2.5)
*P*-value	<0.001	0.212

Univariate Cox Regression model: unadjusted. Multivariate Cox Regression model: adjusted for initial eGFR, initial mean arterial pressure, and initial proteinuria

*Abbreviations*: *CI* Confidence interval, *HR* Hazard ratio, *M0* Mesangial hypercellularity≤0.5, *M1* Mesangial hypercellularity>0.5, *E0* Absence of endocapillary hypercellularity, *E1* Presence of endocapillary hypercellularity, *S0* Absence of segmental glomerulosclerosis, *S1* Presence of segmental glomerulosclerosis, *T0* Tubular atrophy/ interstitial fibrosis 0–25% of cortical area, *T1* Tubular atrophy/ interstitial fibrosis 26–50% of cortical area, *T2* Tubular atrophy/interstitial fibrosis≥50% of cortical area, *C0* Absence of crescents, *C1* Crescents in at least one but < 25% of glomeruli, *C2* Crescents in more than 25% of glomeruli

### Predictive value of lesion M, E and C between immunosuppressive and without immunosuppressive groups

**Fig. 3:**
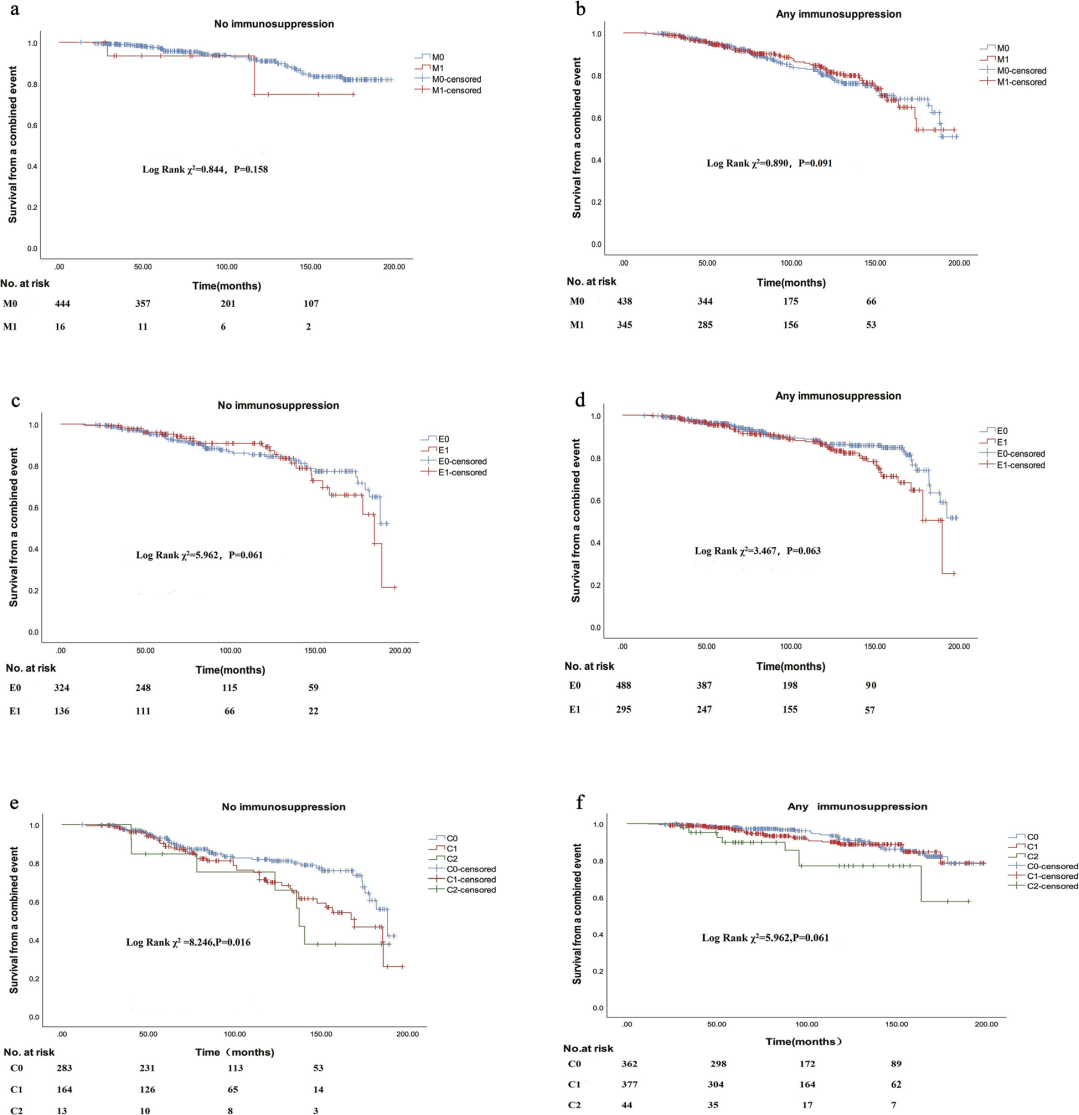
Predictive value of mesangial hypercellularity, endocapillary hypercellularity and cellular/fibrocellular crescents between immunosuppressive and without immunosuppressive groups. **a** Effect of the presence of mesangial hypercellularity on survival from a combined event in patients without immunosuppression. **b** Effect of the presence of mesangial hypercellularity on survival from a combined event in patients with immunosuppression. **c** Effect of the presence of endocapillary hypercellularity on survival from a combined event in patients without immunosuppression. **d** Effect of the presence of endocapillary hypercellularity on survival from a combined event in patients with immunosuppression. **e** Effect of the presence of cellular/fibrocellular crescents on survival from a combined event in patients without immunosuppression. **f** Effect of the presence of cellular/fibrocellular crescents on survival from a combined event in patients with immunosuppression

We further assessed the predictive value of lesions M, E and C in children who received no immunosuppressive treatment to assess their natural predictive value. Children with crescent who didn’t receive immunosuppressive therapy experienced worse survival from the combined event (Fig. 3e), but this difference disappeared after received immunosuppression (Fig. 3f). The predictive value of lesion M and E were not changed by adding immunosuppressive treatment (Fig. 3a-d).

## Discussion

This study investigated the clinical and histopathologic predictors of poor prognosis in pediatric patients with IgAN. The median duration from onset to renal biopsy was 12 months in our cohort. An early diagnosis seemed to be the primary reason for a low frequency of chronic and severe lesions such as lesion S, T and C. In our cohort, we confirmed that S and T were independent risk factors associated with renal outcomes. The lesion C enhanced the ability to predict progression only in those who did not receive immunosuppression. Lesion M and E were not significant variables, which may weaken their predictive values because of the low percentage in the cohort. The independent predictive value of pathology MEST-C score was reduced by immunosuppressive therapy.

Our results suggested that patients with severe pathological lesions (e.g. S、T、C) were associated with lower eGFR, higher blood pressure and higher proteinuria, which were consistent with other findings [7–9]. Glomerular hypertension may mediate progressive renal damage by leading to glomerular hyperfiltration and glomerular enlargement [7]. For the control target of blood pressure in IgAN, the KDIGO guidelines [8] pointed out that when proteinuria > 0.3 g/day, the recommended target blood pressure (BP) was < 130/80 mmHg, and when proteinuria > 1 g/d, the recommended target BP was < 125/75 mmHg. Bellur et al. [9] showed that S was strongly associated with proteinuria and lower eGFR levels, which was consistent with our conclusion. Previous studies [10] have shown that T was an independent risk factor for poor renal prognosis and associated with BP. Some scholars [11, 12] had found that the level of eGFR was lower in patients with IgAN with extensive crescent formation, and there was a negative correlation between eGFR and the proportion of crescents. Thus, it can be concluded that the most critical risk factors for the progression of IgAN (proteinuria, eGFR, MAP) are significantly correlated with the pathological damage found by renal biopsy, which reflects the value of the combination of clinical and pathological risk factors in judging the prognosis of IgAN children.

The lesion M was not a significant risk for renal outcome in our cohort. We speculated that it might be difficult to address its value because of its low prevalence in our study. But the value of lesion M as an independent risk for progression is debated. On the one hand, the VALIGA cohort [13] and a Chinese adult cohort [14] confirmed M lesion as a significant factor for progression, but on the other Shima et al. [15] reported that M had lost predictive value in patients receiving immunosuppressive therapy. By and large, the presence of M lesion appears to have a negligible correlation with renal outcomes.

The lesion E, observed in 35% of the children, did not independently predict clinical outcome in the whole cohort. This was highly consistent with the findings in the Oxford classification cohort [16]. However, two studies in which patients did not receive immunosuppressive therapy [17, 18] reported that E1 was independently associated with more rapid loss of renal function and worse renal survival, which indirectly suggested that proliferative lesions were treatment-responsive. We conjectured that the widespread use of immunosuppressants might have influenced the absence of correlation between E lesion and outcome.

Our data showed relevance between lesion S and renal prognosis, which further confirmed that S was a particular index to judge the prognosis. Many data from the children’s cohort have proved the independent predictive value of S lesion. Children’s group from France confirmed that lesion S was the only histological variable predicting a decline in renal function and was not associated with clinical data at the time of renal biopsy and whether they received immunosuppressive therapy [19]. Studies [20] have revealed S lesion develops from the organization of previous segmental necrotizing or endocapillary inflammatory lesions or in response to podocyte injury and detachment. Therefore, it has also been suggested that in children with active glomerular lesions, special attention needs to be paid to the relationship between lesion S and M and E.

The lesion T was confirmed to be associated with renal failure, which was accord with almost all previous adult validation studies [4, 8, 10]. This may not be surprising because T lesion represents a more chronic and late stage of IgAN renal damage. However, most children validation studies, such as Japan cohort [15], Sweden cohort [21] and VALIGA cohort [13], failed to confirm that T lesion could maintain independent predictive value in children. Only the cohort from China by Le et al. [22] and our cohort confirmed that T lesion has independent predictive value in children population. This difference may be due to only a small proportion of children arrived at a composite terminal during the follow-up in these child studies [13, 15, 21].

In our research, particular interest was given to children with lesion C as they had a predictive value (HR 2.1, 95% *CI* 1.5–2.8) in the univariate analysis, although it did not retain its significance in the multivariate analysis. C lesion was seen in 49% of the children, however, with 28% having crescents in<10% of glomeruli. At the same time, a higher percentage of children with C were treated with immunosuppression than children without this lesion. Overall, crescents predicted a higher risk of a combined event, although this remained significant only in children not receiving immunosuppression. Thus, crescents in a minority of glomeruli may represent a lesion reversible by immunosuppressive therapy. Our findings suggest that children whose biopsies show these active lesions should be considered for immunosuppressive treatment, which was consistent with a multicenter study [23].

The validation differences among the above different child cohorts are mainly related to the regional and ethnic differences in IgAN, the selection criteria, follow-up time and treatment measures of each study, which emphasizes the need to generate a large database for IgAN children to address the problem of insufficient statistical power due to the small number of progressive cases, especially the relatively short follow-up period.

Our cohort validated the significance of the Oxford classification in a large number of Chinese IgAN children. A comprehensive analysis of the renal pathological features and clinical conditions represented in the cohort suggests that Oxford classification must be considered in conjunction with clinical features (including proteinuria levels and eGFR values) and treatment given after renal biopsy. This also suggests that treatment operations after biopsy may regulate some pathological risk factors. To explore the risk factors and their impact on disease progression by studying the clinical and pathological features of IgAN, the level of diagnosis and treatment of IgAN will ultimately be improved.

The limitations of this study must be recognized. First, retrospective design makes the control of measured variables difficult. Second, our results may not be extrapolated to other ethnic groups due to geographical variability in IgAN outcomes. Final, due to the limitations of retrospective studies, not all children were treated with RASB, which may weaken the rigour of the study. However, some features of this study may increase the strength of these findings, including the broad set of data collected over many years and long-term follow-up by the same team with a well-established clinical protocol, as well as the careful re-evaluation of all renal biopsies by two expert pathologist blinded to clinical data and outcome.

## Conclusions

In summary, this study showed that T and S lesions were independently linked to poor renal outcome in Chinese IgAN children. In contrast, C lesion showed significant association with prognosis only in children received no immunosuppressive treatment. M and E lesions appeared to be unrelated to renal prognosis.

## Data Availability

The datasets used and analyzed in this study are available from the first author and corresponding author on reasonable request.

## Abbreviations

C: Crescent
CI: Confidence intervals
eGFR: Estimated glomerular filtration rate
E: Endocapillary proliferation
ESRD: End-stage renal disease
GC: Glucocorticoid
IgAN: IgA Nephropathy
HR: Hazard ratio
IQR: Interquartile range
M: Mesangial proliferation
MAP: Mean arterial pressure
KDIGO: The Kidney Disease Improving Global Outcomes
RASB: Renin angiotensin aldosterone system blockade
S: Segmental sclerosis/adhesion lesion
T: Tubular atrophy/interstitial fibrosis
VALIGA: European Validation Study Of The Oxford Classification Of IgA Nephropathy
